# Effect on Insulin, Glucose and Lipids in Overweight/Obese Australian Adults of 12 Months Consumption of Two Different Fibre Supplements in a Randomised Trial

**DOI:** 10.3390/nu9020091

**Published:** 2017-01-29

**Authors:** Sebely Pal, Suleen Ho, Roland J. Gahler, Simon Wood

**Affiliations:** 1 School of Public Health, Curtin University, Perth 6845, Australia; suleen.ho@curtin.edu.au; 2 Factors Group Research, Burnaby, BC V3N 4S9, Canada; rgahler@naturalfactors.com; 3 Food, Nutrition and Health Program, University of British Columbia, Vancouver, BC V6T 1Z4, Canada; simonwood@shaw.ca

**Keywords:** obesity, PGX, psyllium, cholesterol, insulin

## Abstract

Higher fibre intakes are associated with risk reduction for chronic diseases. This study investigated the effects of supplementation with PolyGlycopleX^®^ (PGX), a complexed polysaccharide, on insulin, glucose and lipids in overweight and obese individuals. In this double-blind 12 months study, participants were randomised into three groups: control (rice flour); PGX or psyllium (PSY). Participants followed their usual lifestyle and diet but consumed 5 g of their supplement before meals. Insulin was significantly lower in the PGX and PSY groups compared to control at 3 and 6 months and in the PSY group compared to control at 12 months. Serum glucose was significantly lower in the PGX group at 3 months compared to control. Total cholesterol was significantly lower in the PGX and PSY groups compared to control at 3 and 6 months. High density lipoprotein (HDL) cholesterol was significantly increased in the PGX group compared to control at 12 months. low density lipoprotein (LDL) cholesterol was significantly lower in the PGX group at 3 and 6 months compared to control and in the PSY group at 3 months compared to control. A simple strategy of fibre supplementation may offer an effective solution to glucose, insulin and lipid management without the need for other nutrient modification.

## 1. Introduction

Previous studies have consistently shown that higher fibre intakes are correlated with lower body weight, body mass index (BMI), waist circumference [1,2] and improved plasma lipid profiles [3,4,5,6,7,8,9,10,11,12], glycaemia and insulinaemia [13], indicating benefits and risk reduction for metabolic syndrome, cardiovascular disease (CVD) and type 2 diabetes.

While the benefits of a high fibre diet are well known, increasing fruit and vegetable intake to meet the recommended intake of fibre is difficult for many [14]. Present estimations of dietary fibre intake in Australian, Canadian, European and American adults is approximately 15–25 g/day [3,15], which is below the current recommendations for adults in Australia, Canada, Europe and the USA of 25–30 g/day [16]. Therefore, fibre supplements can provide a cost effective and easy alternative method for increasing the fibre content of a diet without the need for other major nutrient modifications.

PolyGlycopleX (PGX) has been shown to have lipid lowering effects in healthy subjects [17] as well as in overweight and obese adults [18]. In a clinical trial with healthy subjects, 25 males and 29 females, mean age of 31.6 ± 10.5 years entered the study [17] and consumed 2.5 g of PGX twice a day as part of two main meals (breakfast and/or lunch, and/or dinner) for the first seven days, followed by 5 g PGX for the last 14 days of the study. The control product was a skimmed milk powder. PGX’s effects on decreasing total and low density lipoprotein (LDL) cholesterol levels in the study concur with similar reports in the literature [19]. Studies are required to investigate if these benefits are sustainable over a longer duration and also in those who are overweight/obese. Another study with 29 overweight and obese adults, ages 20–65 with a BMI between 25–36 kg/m^2^, consuming 5 g of PGX in 500 mL of water 5–10 min before each meal, 2–3 times a day for 14 days showed a significant reduction of total cholesterol (TC) and LDL levels of 19% and 25% respectively, compared to baseline [18]. However, the supplement was combined with advice for healthy eating, weight loss and exercise so it is difficult to evaluate the effect of the fibre alone on lipids. This was not a randomised controlled trial as there was no control group in this study. Recent human studies have shown that the addition of 2.5 to 5 g of PGX with a meal is highly effective in reducing postprandial glycaemia, lowering the glycaemic index of food [20] and modifying satiety hormones in healthy adults [21]. However, these studies have some limitations; they were acute or short term, mostly conducted in healthy, normal weight participants or combined with lifestyle changes.

Psyllium has been evaluated in various human studies for effects on glucose and insulin homeostasis, body weight, body composition and appetite as well as lipids and lipoproteins [22,23,24,25,26,27,28,29,30]. Psyllium was reviewed in 2012 for its effect on metabolic syndrome [31]. The authors concluded that “Collectively, research to date does support the notion that the consumption of psyllium may provide benefits to many components of the metabolic syndrome”. Psyllium fibre decreased fat absorption in overweight and obese men, but had no effect on postprandial glucose and insulin concentrations [32]. In another study, simply adding a psyllium fibre supplement to a normal diet (10.2 g/day) was sufficient to see improvements in TC and LDL cholesterol but not fasting glucose or insulin concentrations when compared to the control group [22]. However, a meta-analysis [33] found that in type 2 diabetes patients, psyllium fibre significantly improved fasting blood glucose, proportional to loss of glycaemic control.

Apart from these few studies, limited research is available on the effect of PGX fibre on blood insulin, glucose and lipids. Randomised controlled clinical trials are required to verify whether PGX can be used for improving insulin, glucose and lipids in the long term and whether this fibre type is better than other soluble fibres, such as psyllium. Therefore the aim of this study was to investigate the effect of PGX on insulin, glucose and lipid concentrations. Given the effect of PGX on total and LDL cholesterol in healthy weight participants and its considerably higher viscosity, we hypothesise that PGX will have a greater health outcome than psyllium in overweight and obese individuals.

## 2. Materials and Methods

### 2.1. Subjects

Overweight and obese individuals with a body mass index (BMI) between 25–47 kg/m^2^ and aged between 19 and 68 years, were recruited from the community in Perth, Australia (via newspaper and radio). Potential participants were screened by telephone or online questionnaire and attended Curtin University to assess suitability for the study, at which time the details of the study were explained. Exclusion criteria included smoking, lipid lowering medication, use of steroids and other agents that may influence lipid metabolism, use of warfarin, diabetes mellitus, hypo- and hyperthyroidism, cardiovascular events within the last 6 months, psychological unsuitability, major systemic diseases, gastrointestinal problems, proteinuria, liver, renal failure, weight fluctuations over the past 6 months, vegetarianism and participation in any other clinical trials within the last 6 months. This study was approved by and conducted in accordance with the ethical standards of Curtin Human Research Ethics Committee. Written consent was obtained from all participants. ANZCTR number: ACTRN12611000415909.

### 2.2. Study Design

This study is part of a larger trial, which was a randomised, double blind, parallel design study over a 52 week period. Study participants were randomised by the trial sponsors using a Web site (http://www.randomization.com) to one of the three groups (three randomly permuted blocks): the control group who consumed the placebo with their usual diet; the psyllium supplement group (PSY) who consumed a psyllium supplement with their usual diet and a PGX supplement group (PGX) who consumed a PGX supplement with their usual diet. Psyllium is a soluble fibre and PolyGlycopleX (PGX) is a novel, highly viscous functional non-starch polysaccharide complex, with developing viscosity, manufactured from konjac (glucomannan), sodium alginate and xanthan gum by a proprietary process (EnviroSimplex^®^). The fibre supplementation consisted of either 5 g of psyllium (a proprietary psyllium product with the trade name PgxSyl™) (InovoBiologic, Inc., Calgary, AB, Canada) or 5 g of PGX (InovoBiologic, Inc., Calgary, AB, Canada). Placebo consisted of 5 g rice flour. All supplements were artificially sweetened and flavoured. The rice flour provided an appropriate placebo due to its low energy and fibre content and similarity in texture and appearance to the psyllium and PGX supplement. Participants were instructed to take either 5 g of the fibre supplements or placebo, mixed with a minimum of 250 mL water followed by a further 250 mL water, three times daily, 5–10 min before breakfast, lunch and dinner. Extra water was allowed to be taken *ad libitum* during or after the meal if desired and subjects were encouraged to do this. The supplement packages (control, PGX and psyllium) were from a single batch provided by the manufacturer, and appeared identical so that the research assistants and participants were blinded to the type of supplement being consumed. Packages consisted of 5 g doses of the control, PGX or psyllium and were only marked by the participant ID, with the group allocation only known to the trial sponsors to ensure blinding. Quantities of rice flour and psyllium were determined by input, with the amounts weighed and checked by the dispensing and blending department while PGX was analysed according to USP Monograph FCC9 3rd Sup 2015 [34]. All identifiable information from participants was coded to ensure privacy.

The subjects attended a briefing session on how to consume the supplement, complete food records and comply with the study protocol as previously reported [35]. Briefly, the dietary intake over the course of the trial was monitored through the completion of 3-day food diaries at baseline, 12, 26 and 52 weeks. All participants in the control and the fibre supplement groups were asked to maintain their usual dietary intake for the duration of the study. To monitor compliance, all participants were required to complete a diary to record their supplement consumption and asked to return both the empty and unused sachets of the supplements at their visits.

### 2.3. Anthropometry and Body Composition

Measures of body weight, height, waist and hip circumference were undertaken at baseline, 3, 6 and 12 months. Body weight (HBF-514, Omron, Kyoto, Japan) was recorded in light clothing without shoes. Height was measured to the nearest 0.1 cm using a stadiometer without shoes. Waist circumference was measured in the standing position at the narrowest area between the lateral lower rib and the iliac crest. Hip measurement was taken at the largest circumference of the lower abdomen.

### 2.4. Diet and Physical Activity

Participants completed 3-day food and drink diaries at baseline, 3, 6 and 12 months to monitor for changes in food intake. Data were analysed with Foodworks 7 Professional (Xyris Software, Brisbane, Australia), based on data from the AUSNUT database. Participants also completed the International Physical Activity Questionnaire (IPAQ) at the same time points.

### 2.5. Measurements of Lipids, Glucose and Insulin Levels

Participants attended clinical rooms at Curtin University Bentley, after a 10–12 h fast, for baseline measurements. Fasting blood samples (20 mL) were drawn by venipuncture. The collection of fasting blood samples was repeated at 3, 6 and 12 months. Serum tubes were allowed to clot and blood samples were centrifuged at 2500 rpm at 4 °C for 10 min using a Hettich Rottina 48R centrifuge. Serum and plasma were then aliquoted and samples stored at −80 °C and analysed after study completion.

Serum triglyceride and total cholesterol was measured by enzymatic colorimetric kits (TRACE Scientific Ltd., Melbourne, Australia). Serum HDL cholesterol was determined after precipitation of apoB (apolipoprotein B)-containing lipoproteins with phosphotungstic acid and MgCl_2_ (magnesium chloride); the supernatant containing the HDL cholesterol was determined by enzymatic colorimetry (TRACE Scientific Ltd., Melbourne, Australia). Serum LDL cholesterol was determined by using the Friedewald equation [36]. Non-esterified fatty acid (NEFA) was determined using WAKO NEFA C kit (Osaka, Japan). ApoB was analysed using an ELISA kit obtained from Mabtech AB (Nacka Strand, Stockholm, Sweden).

Plasma glucose concentrations were measured using the Randox glucose GOD-PAP kit (Antrim, UK), according to the manufacturer’s instructions. Plasma insulin was measured by an ELISA kit (Alpha Diagnostics International, San Antonio, TX, USA). HOMA2-IR (homeostasis model assessment of insulin resistance) was used to assess insulin resistance from fasting glucose and insulin concentrations using a computer model [37].

### 2.6. Statistical Analysis

A sample size of 24 subjects per group was predicted to provide sufficient power (80%) to detect a 3% difference in weight before and after treatment within a group. We recruited a total of 53 subjects per group to accommodate for 50% dropouts. Calculations were based on an average mean weight of 80 kg and a standard deviation of 5% within a group on all eligible subjects. Statistical analysis was undertaken using SPSS 22 for Windows (SPSS Inc., Chicago, IL, USA). Data were expressed as mean (±SD or SEM) and assessed for normality to ensure that the assumptions of the analysis were met. Baseline differences between groups were analysed with one-way ANOVA. The data were analysed using general linear models with baseline value covariates. If significant between–groups effects were present, post hoc comparisons between the treatment groups was made using the least significant difference (LSD) method. Statistical significance was considered at *p* < 0.05. Intention–to–treat analysis was also carried out with missing data replaced with the last observation carried forward.

## 3. Results

### 3.1. Participants

**Figure 1:**
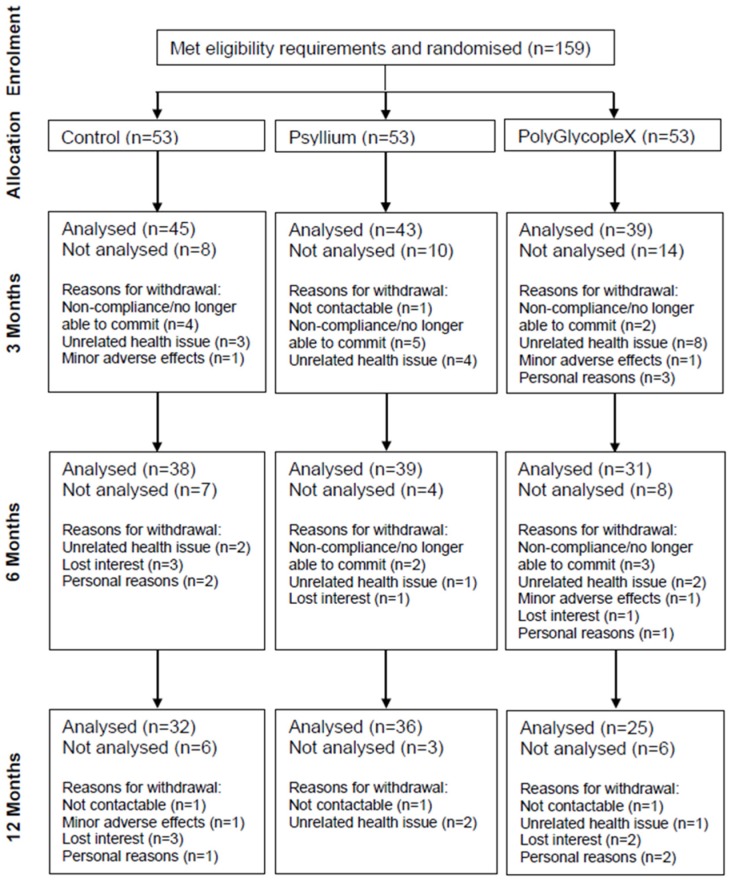
Participant flow diagram.

The 159 participants (19 to 68 years) who met the eligibility criteria were randomised to one of three groups (Control, PSY, PGX) by assignment of an ID number and the corresponding numbered supplement. Participant flow through the study can be seen in Figure 1. Although the PGX group had the highest attrition rate, it was generally due to factors unrelated to the study. A total of 127 participants (54 male, 73 female) completed at least 3 months of the study and were included in the analysis (45 in Control (24 male), 43 in PSY (15 male) and 39 in PGX (15 male)). Thirty-two participants withdrew before 3 months and were excluded from analysis due to non-compliance, unrelated health issues, minor adverse effects and personal reasons. A total of 108 participants at 6 months (38 in Control, 39 in PSY and 31 in PGX) and 93 participants at 12 months (32 in Control, 36 in PSY and 25 in PGX) were analysed. Results for intention–to–treat analysis for the primary outcome variables can be found in supplementary files.

### 3.2. Baseline Characteristics

There were no significant differences at baseline between groups for major characteristics, energy intake, fibre intake, lipids, insulin or glucose (Table 1).

### 3.3. Diet

The dietary analysis can be seen in Table 2. When examining differences between groups, energy intake was significantly lower compared to control at 3 months and 6 months in the PGX and PSY groups and at 12 months only the PGX group demonstrated significantly lower energy intake compared to the control. Carbohydrate intake was significantly lower at 3 months and 6 months in the PGX and PSY groups compared to control. Fat intake and protein intake were significantly lower compared to control at 3 months in the PGX and PSY groups and at 6 months in the PGX group compared to control.

### 3.4. Physical Activity

Physical activity levels did not significantly change from baseline within any groups and there were no significant differences between groups at any time point (Table 3).

### 3.5. Lipids

**Figure 2:**
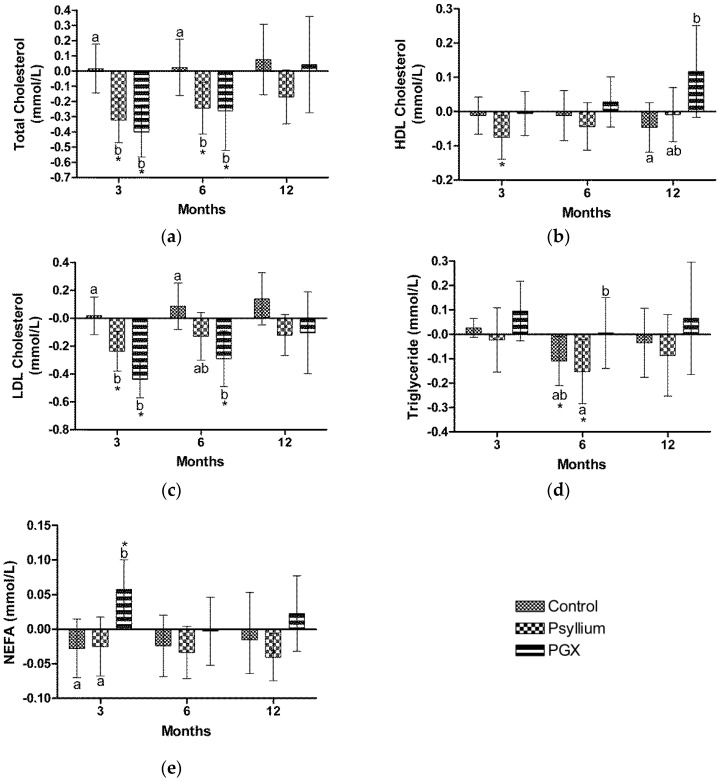
Changes in fasting blood lipids during 12 months of fibre supplementation (**a**) Total Cholesterol; (**b**) high density lipoprotein (HDL); (**c**) low density lipoprotein (LDL); (**d**) Triglyceride; (**e**) NEFA (non-esterified fatty acid). Values are mean ± 95% CI with baseline as a covariate. * indicates within group differences compared to baseline. Different letters represent significant differences between groups *p* < 0.05.

Total cholesterol was significantly lower in the PGX group at 3 months (−8%, *p* < 0.001) and 6 months (−5.1%, *p* = 0.048) compared to baseline, as shown in Figure 2A. Total cholesterol was significantly lower in the PSY group at 3 months (−6.5%, *p* < 0.001) and 6 months (−4.8%, *p* = 0.006) compared to baseline. Total cholesterol was significantly lower at 3 months in the PGX (−8.2%, *p* < 0.001) and PSY (−7%, *p* = 0.001) groups and at 6 months in the PGX (−5.5%, *p* = 0.047) and PSY (−5.3%, *p* = 0.042) groups compared to control. There were no significant differences in total cholesterol between PSY and PGX groups at 3, 6 or 12 months.

High density lipoprotein (HDL) cholesterol was significantly lower in the PSY group at 3 months (−5.5%, *p* = 0.022) compared to baseline, as shown in Figure 2B. HDL was significantly higher at 12 months in the PGX group (11.5%, *p* = 0.019) compared to control. There were no significant differences in HDL between control and PSY or between PSY and PGX groups at 3, 6 or 12 months.

LDL was significantly lower in the PGX group at 3 months (−13.7%, *p* < 0.001) and 6 months (−9.1%, *p* = 0.006) compared to baseline, shown in Figure 2C. LDL cholesterol was significantly lower in the PSY group at 3 months (−7.8%, *p* = 0.002) compared to baseline. LDL was significantly lower compared to control at 3 months in the PGX (−13.9%, *p* < 0.001) and PSY (−8.1%, *p* = 0.007) groups and at 6 months in the PGX (−11%, *p* = 0.006) group compared to control. There were no significant differences in LDL between PSY and PGX groups at 3, 6 or 12 months.

Triglyceride was significantly lower in the control group at 6 months (−7.6%, *p* = 0.033) compared to baseline (Figure 2D). TG was significantly lower in the PSY group at 6 months (−12.7%, *p* = 0.023) compared to baseline.

Non-esterified fatty acid (NEFA) was significantly lower in the PSY group at 12 months (−9.5%, *p* = 0.021) compared to baseline, as shown in Figure 2E. NEFA was significantly higher in the PGX group at 3 months (15.7%, *p* = 0.009) compared to baseline. NEFA was significantly higher in the PGX group compared to control (16.9%, *p* = 0.027) and PSY (18.2%, *p* = 0.017) at 3 months. There were no significant differences in NEFA between control and PSY at 3, 6 or 12 months.

ApoB did not significantly change within any of the study groups. There were no significant differences in apoB between control, PSY or PGX groups at 3, 6 or 12 months (data not shown).

### 3.6. Insulin

**Figure 3:**
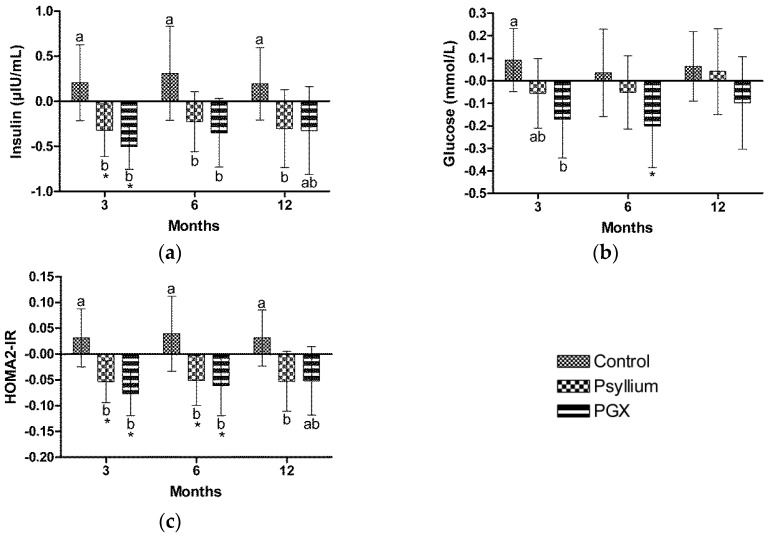
Changes in fasting blood parameters during 12 months of fibre supplementation (**a**) Insulin, (**b**) Glucose; (**c**) HOMA2-IR. Values are mean ± 95% CI with baseline as a covariate. * indicates within group differences compared to baseline. Different letters represent significant differences between groups *p* < 0.05.

Insulin was significantly lower in the PGX group at 3 months (−7.6%, *p* < 0.001) compared to baseline (Figure 3A). Insulin was significantly lower in the PSY group at 3 months (−5.5%, *p* = 0.032) compared to baseline. Insulin was significantly lower compared to control at 3 months in the PGX (−9%, *p* = 0.008) and PSY (−9.4%, *p* = 0.004) groups, at 6 months in PGX (−9.8%, *p* = 0.038) and PSY (−9.1%, *p* = 0.040) groups compared to control and at 12 months in the PSY group (−9.4%, *p* = 0.029) compared to control. There were no significant differences in insulin between PSY and PGX groups at 3, 6 or 12 months.

### 3.7. Glucose

Glucose was significantly lower in the PGX group at 6 months (−3.9%, *p* = 0.033) compared to baseline, while the decrease at 3 months did not reach significance (−3.4%, *p* = 0.053) (Figure 3B). Glucose was significantly lower compared to control at 3 months in the PGX (−4.8%, *p* = 0.019) group. There were no significant differences in glucose between control and PSY or between PSY and PGX groups at 3, 6 or 12 months.

### 3.8. Homeostasis Model Assessment of Insulin Resistance

The HOMA2-IR score was significantly lower in the PGX group at 3 months (−9%, *p* = 0.001) and 6 months (−7.3%, *p* = 0.039) compared to baseline (Figure 3C). HOMA2-IR was significantly lower in the PSY group at 3 months (−7%, *p* = 0.011) and 6 months (−6.7%, *p* = 0.037) compared to baseline. HOMA2-IR was significantly lower compared to control at 3 months in the PGX (−10.8%, *p* = 0.005) and PSY (−10.8%, *p* = 0.001) groups, at 6 months in PGX (−11.9%, *p* = 0.033) and PSY (−11.9%, *p* = 0.018) groups compared to control and at 12 months in the PSY group (−11%, *p* = 0.011) compared to control. There was a trend for HOMA2-IR to be lower in the PGX group compared to control at 12 months but this was not significant (−8.5%, *p* = 0.068). There were no significant differences in HOMA2-IR score between PSY and PGX groups at 3, 6 or 12 months.

### 3.9. Adverse Events

Minor adverse events were gastrointestinal related (e.g., flatulence, diarrhoea) with four withdrawing from the study, two in the PGX group and two in the control group. The PSY supplement was better tolerated and participants did not report any adverse effects.

## 4. Discussion

Previous epidemiological and cohort studies have consistently revealed that higher fibre intakes are correlated with lower body weight, BMI, waist circumference [1,2], and improved plasma lipid profiles [3,4,5,6,7,8,9,10,11,12], glycaemia and insulinaemia [13], indicating the benefits and risk reduction for the metabolic syndrome, CVD and type 2 diabetes. Given that individuals find it difficult to eat the required amounts of fibre by increasing fruit and vegetable intake, it was hypothesised that fibre supplements can provide similar health benefits compared with increased dietary fibre intake. Therefore, this study investigated the effects of 15 g of PGX or psyllium compared to control (rice flour) supplementation for one year on lipids, insulin and glucose. Specifically, both the PGX and PSY groups demonstrated significant reductions at 3 months in fasting concentrations of total cholesterol by 8.2% and 7% respectively, LDL cholesterol by 13.9% and 8.1% respectively, insulin by 9% and 9.4% respectively and HOMA2-IR score by 11.9%, compared to control group. Only the PGX intervention improved fasting glucose and HDL cholesterol during the study.

Our results herein are supported by recent human and animal studies which suggest that psyllium fibre supplementation may provide cardiovascular benefits [22,31]. The effect of PGX on decreasing total and LDL cholesterol levels in this study concurs with similar reports in the literature describing the effects of viscous dietary fibre on lowering serum cholesterol levels. Carabin et al. [17] conducted a clinical trial with healthy subjects, 25 males and 29 females, mean age of 31.6 ± 10.5 years who consumed 2.5 g of PGX packaged with cereal and yoghurt twice a day as part of two main meals (breakfast and/or lunch, and/or dinner), for the first seven days, followed by 5 g PGX twice a day for the last 14 days of the study. The control product was a skimmed milk powder. Investigators observed significantly lower total and LDL cholesterol in the test group compared to the control group at day 8 (9.4% vs. 3.3% for total and 10.4% vs. 2.5% for LDL cholesterol, respectively) and at day 22 (14.1% vs. 8.3% for total and 16.6% vs. 8.2% for LDL cholesterol, respectively). The differences between groups was similar to the magnitude observed in our study. Reimer et al. [38] observed the effects of 14 weeks of short-term PGX supplementation in adults with abdominal obesity and also observed significant reductions in total and LDL cholesterol. In a pre-post study by Lyon & Reichert [18], 29 overweight and obese adults with a BMI between 25–36 kg/m^2^, consumed 5 g of PGX in 500 mL of water 5–10 min before each meal, 2–3 times a day for 14 days and showed a significant reduction of total cholesterol and LDL cholesterol levels of 19% and 25% respectively, compared to baseline. As the supplement intervention in this latter study was combined with lifestyle changes, the decreases in cholesterol levels were greater than those observed in our current study. Although neither of the studies described above reported changes in HDL after PGX supplementation, we only observed this effect at 12 months, indicating a possible long term effect that has not been demonstrated previously. When looking at the effect of psyllium, another study [22] also found that simply adding a psyllium fibre supplement to a normal diet (10.2 g/day) was sufficient to see improvements in total cholesterol (−21%) and LDL cholesterol (−22%) at 12 weeks compared to control. Similar findings for psyllium have also been reported in various meta-analyses [39].

Animal studies have demonstrated a beneficial effect of PGX supplementation on glucose control and fasting insulin [40]. While many human studies have examined the short-term postprandial glucose response to PGX supplementation [20,41,42], few have looked at the long term effects. Lyon & Reichert [18] assessed changes to fasting insulin and glucose in their study. The PGX and lifestyle intervention caused a 6.96% reduction in fasting glucose and a 27.26% reduction in fasting insulin which is in agreement with our PGX intervention findings. Psyllium has been previously shown to improve glucose and insulin response [31,33]. In a study by Ziai et al. [23], psyllium was taken in combination with medication and a significant reduction in fasting glucose was observed. Gibb et al. [33] reported that psyllium improved glycaemic control proportional to loss of glycaemic control, which may explain why our non-diabetic psyllium group did not demonstrate any significant improvements to fasting blood glucose concentrations.

Due to its high viscosity, PGX swells in the stomach and increases feelings of fullness [23,42,43,44,45]. Psyllium has also been shown to increase fullness [29] but has a far lower viscosity than PGX [18]. This characteristic may have caused participants to decrease their food intake, which then lead to significant weight loss [46]. The changes to blood lipids and insulin observed are likely due to the changes in dietary intake and weight loss observed as well as the possible effect of PGX slowing gastric emptying and absorption of nutrients in the small intestine [47].

When comparing the PGX and PSY groups in the current study, there were no significant differences between them except for a lower concentration of TG at 3 months in the PSY group compared to the PGX group. However, when examining differences compared to control, HDL cholesterol was significantly higher in the PGX group at 12 months but not in the PSY group. LDL cholesterol was significantly lower in the PGX group at 3 and 6 months compared to control whereas LDL cholesterol was only significantly lower in the PSY group at 3 months compared to control. Glucose was significantly lower in the PGX group at 3 months but not in the PSY group. Intent-to-treat analysis was carried out but the outcome results were no different from the results presented. In this regard, the PGX group performed better overall than the PSY group and elicited more health benefits over the 12 months intervention period.

One of the strengths of this study was the duration, with a 12 months intervention period. Comparable studies have only been conducted for 14 weeks. This allowed us to investigate the long term effects of the fibre supplements, especially weight maintenance. This was a double-blinded study and supplements were packed in identical foil sachets. Investigators were not aware which participants were taking which supplement; however, due to the different characteristics of the supplements, participants may have been able to guess if they were taking a fibre supplement or the control, which is a limitation. The intervention was not combined with any other lifestyle modification advice, thus it would be simple for consumers to incorporate into their lifestyle or there could be added benefits if the supplements were combined with healthy lifestyle advice. Other study limitations include the use of a generally healthy population. We did not specifically recruit participants with elevated lipid, insulin or glucose concentrations which would have limited our ability to detect significant improvements. The majority of participants were women and hormonal changes over the 12 months may have impacted on lipids levels.

## 5. Conclusions

It is thought the high viscosity of PGX caused participants to decrease their food intake, which then lead to significant weight loss, lipid, insulin, and glucose reductions. Taking a fibre supplement before meals was a relatively easy task for people to incorporate into their daily routine and would be a simple intervention to implement. We observed similar results between PGX and PSY supplements when compared to the control group, however the PGX supplement was superior in terms of increased HDL cholesterol and decreased fasting blood glucose. Therefore, regular consumption of a PolyGlycopleX or a psyllium supplement is a simple and effective method to improve blood lipids, insulin and glucose control in overweight or obese people and may lead to risk reduction for metabolic syndrome, CVD and type 2 diabetes.

## Figures and Tables

**Table 1:** Baseline characteristics.

	Control (*n* = 45)	PSY (*n* = 43)	PGX (*n* = 39)	*p*
Gender (Male/Female)	24/21	15/28	15/24	
Age (year)	49.82 ± 11.75	49.93 ± 11.04	47.87 ± 12.08	
Height (cm)	171.68 ± 10.04	169.16 ± 10.52	169.76 ± 10.51	
Weight (kg)	94.69 ± 17.05	91.17 ± 14.74	96.24 ± 18.02	0.365
BMI (kg/m^2^)	32.01 ± 4.2	31.74 ± 3.22	33.25 ± 4.3	0.189
Waist (cm)	103.12 ± 11.02	101.17 ± 9.76	105.97 ± 12.72	0.154
Hip (cm)	112.87 ± 9.04	114.63 ± 8.63	115.71 ± 9.37	0.414
Waist Hip Ratio	0.91 ± 0.08	0.88 ± 0.08	0.92 ± 0.1	0.158
TC (mmol/L)	5 ± 0.85	4.93 ± 1.06	5.02 ± 0.85	0.904
HDL (mmol/L)	1.33 ± 0.37	1.36 ± 0.38	1.28 ± 0.26	0.563
LDL (mmol/L)	3.02 ± 0.87	3.04 ± 0.96	3.2 ± 0.77	0.592
TG (mmol/L)	1.44 ± 1.12	1.18 ± 0.67	1.2 ± 0.56	0.269
Insulin (μIU/mL)	6.11 ± 1.43	5.87 ± 1.64	6.56 ± 1.94	0.172
Glucose (mmol/L)	4.94 ± 0.73	4.79 ± 0.52	4.99 ± 0.56	0.302

Values are mean ± SD. PSY (Psyllium), PGX (PolyGlycopleX), TC (Total Cholesterol), TG (Triglyceride). *p* values are differences between groups.

**Table 2:** Dietary intake during 12 months of fibre supplementation.

Variable		3 Months	*n*	*P*	6 Months	*n*	*P*	12 Months	*n*	*p*
Energy (kJ/day)	CTR	9013.1 ± 223.6 ^a^	39	0.641	8803.3 ± 282.9 ^a^	34	0.928	8218 ± 295.4 ^a^	31	0.111
	PSY	7272.3 ± 218 ^b^	41	<0.001	7539.2 ± 278.8 ^b^	35	<0.001	7657.1 ± 277.9 ^a,b^	35	0.001
	PGX	7556.3 ± 243.1 ^b^	33	0.001	7453.7 ± 323.5 ^b^	26	0.009	7315.3 ± 342.4 ^b^	23	0.012
CHO (g/day)	CTR	212.6 ± 7.7 ^a^	39	0.827	213.4 ± 10 ^a^	34	0.787	200.5 ± 8.4	31	0.212
	PSY	181 ± 7.5 ^b^	41	0.002	164.7 ± 9.8 ^b^	35	0.002	183.9 ± 7.9	35	0.015
	PGX	175.3 ± 8.4 ^b^	33	0.003	177.2 ± 11.4 ^b^	26	0.032	176.3 ± 9.7	23	0.012
Fat (g/day)	CTR	83.8 ± 3.2 ^a^	39	0.907	82.3 ± 4.3 ^a^	34	0.887	73 ± 4	31	0.066
	PSY	66.9 ± 3.1 ^b^	41	<0.001	72.9 ± 4.2 ^a,b^	35	0.147	73 ± 3.8	35	0.070
	PGX	72.2 ± 3.4 ^b^	33	0.055	69.1 ± 4.9 ^b^	26	0.047	66 ± 4.7	23	0.020
Total Fibre (g/day)	CTR	24.2 ±1.2 ^a^	39	0.599	22.1 ± 1 ^a^	34	0.790	21.6 ± 1.3 ^a^	31	0.613
	PSY	36.4 ± 1.2 ^b^	41	<0.001	35.1 ± 0.9 ^b^	35	<0.001	36.6 ± 1.3 ^b^	35	<0.001
	PGX	36.6 ± 1.3 ^b^	33	<0.001	34.1 ± 1.1 ^b^	26	<0.001	36.1 ± 1.6 ^b^	23	<0.001

Values are mean ± SEM with baseline as a covariate. Different letters in superscript represent significant differences between groups *p* < 0.05. *p* Values are within group differences compared to baseline. CHO (carbohydrate), CTR (control), PGX (PolyGlycopleX), PSY (psyllium).

**Table 3:** Physical activity during 12 months of fibre supplementation.

	3 Months	Mean Change	*n*	*P*	6 Months	Mean Change	*n*	*P*	12 Months	Mean Change	*n*	*p*
CTR	2802.4 ± 449.3	−130.5	29	0.837	2868.2 ± 454.7	−141.2	32	0.823	3294.5 ± 525.6	415.4	30	0.569
PSY	2933 ± 443.6	620.8	30	0.345	3009.3 ± 433.2	187	35	0.876	2879.1 ± 497.8	609.6	33	0.526
PGX	2181.7 ± 470.6	751.3	27	0.254	2681.1 ± 495.5	328.2	27	0.711	2684.9 ± 619.1	194.3	22	0.886

Values are mean kJ/day ± SEM with baseline as a covariate. Mean change from baseline. *p* Values are within group differences compared to baseline.

## Supplementary Materials

The following are available online at http://www.mdpi.com/2072-6643/9/2/091/s1, Table S1: Lipids, Glucose and Insulin levels during 12 months of fibre supplementation (intention to treat analysis).
