# Supplementary material for: Effect on Insulin, Glucose and Lipids in Overweight/Obese Australian Adults of 12 Months Consumption of Two Different Fibre Supplements in a Randomised Trial
Source: Nutrients. 2017 Jan 29;9(2):91. doi: 10.3390/nu9020091 (PMC5331522; doi:10.3390/nu9020091)
Supplement: Supplementary file 1 [file nutrients-09-00091-s001.docx]

Supplementary Materials: Effect on Insulin, Glucose and Lipids in Overweight/Obese Australian Adults of 12 Months Consumption of Two Different Fibre Supplements in a Randomised Trial

Sebely Pal, Suleen Ho, Roland J. Gahler and Simon Wood

**Table S1.** Lipids, Glucose and Insulin levels during 12 months of fibre supplementation (intention to treat analysis).

|  | | | | | | | | | **Between Group *P* Values** | | | |
| --- | --- | --- | --- | --- | --- | --- | --- | --- | --- | --- | --- | --- |
| **Variable** |  | ***n*** | **3 Months** | ***p*** | **6 Months** | ***p*** | **12 Months** | ***p*** |  | **3 Months** | **6 Months** | **12 Months** |
| Total Cholesterol | CTR | 45 | 5 ± 0.07 ^a^ | 0.900 | 5.03 ± 0.08 ^a^ | 0.657 | 5.05 ± 0.09 ^a^ | 0.509 | CTR vs. PSY | 0.001 | 0.010 | 0.036 |
|  | PSY | 43 | 4.65 ± 0.07 ^b^ | <0.001 | 4.71 ± 0.09 ^b^ | 0.004 | 4.78 ± 0.09 ^b^ | 0.022 | CTR vs. PGX | <0.001 | 0.006 | 0.176 |
|  | PGX | 39 | 4.59 ± 0.08 ^b^ | <0.001 | 4.68± 0.09 ^b^ | 0.005 | 4.87 ± 0.1 ^a,b^ | 0.292 | PSY vs. PGX | 0.597 | 0.790 | 0.485 |
| HDL | CTR | 45 | 1.31 ± 0.03 | 0.665 | 1.31 ± 0.03 | 0.760 | 1.28 ± 0.03 | 0.227 | CTR vs. PSY | 0.145 | 0.629 | 0.381 |
|  | PSY | 43 | 1.26 ± 0.03 | 0.022 | 1.29 ± 0.03 | 0.268 | 1.33 ± 0.03 | 0.921 | CTR vs. PGX | 0.889 | 0.777 | 0.132 |
|  | PGX | 39 | 1.31 ± 0.03 | 0.751 | 1.3 ± 0.03 | 0.631 | 1.36 ± 0.04 | 0.301 | PSY vs. PGX | 0.206 | 0.853 | 0.516 |
| LDL | CTR | 45 | 3.08 ± 0.06 ^a^ | 0.878 | 3.14 ± 0.07 ^a^ | 0.421 | 3.16 ± 0.08 ^a^ | 0.279 | CTR vs. PSY | 0.009 | 0.029 | 0.036 |
|  | PSY | 43 | 2.84 ± 0.07 ^b^ | 0.002 | 2.9 ± 0.08 ^b^ | 0.050 | 2.92 ± 0.08 ^b^ | 0.043 | CTR vs. PGX | <0.001 | 0.001 | 0.017 |
|  | PGX | 39 | 2.66 ± 0.07 ^b^ | <0.001 | 2.77 ± 0.08 ^b^ | <0.001 | 2.88 ± 0.08 ^b^ | 0.038 | PSY vs. PGX | 0.072 | 0.255 | 0.712 |
| Triglyceride | CTR | 45 | 1.33 ± 0.06 | 0.694 | 1.28 ± 0.06 | 0.611 | 1.34 ± 0.07 ^a,b^ | 0.617 | CTR vs. PSY | 0.269 | 0.112 | 0.071 |
|  | PSY | 43 | 1.24 ± 0.06 | 0.723 | 1.15 ± 0.06 | 0.090 | 1.16 ± 0.07 ^a^ | 0.225 | CTR vs. PGX | 0.732 | 0.677 | 0.797 |
|  | PGX | 39 | 1.36 ± 0.06 | 0.123 | 1.31 ± 0.06 | 0.464 | 1.36 ± 0.07 ^b^ | 0.192 | PSY vs. PGX | 0.157 | 0.051 | 0.045 |
| Insulin | CTR | 45 | 6.35 ± 0.14 ^a^ | 0.302 | 6.45 ± 0.17 ^a^ | 0.187 | 6.41 ± 0.17 ^a^ | 0.169 | CTR vs. PSY | 0.004 | 0.020 | 0.008 |
|  | PSY | 43 | 5.75 ± 0.15 ^b^ | 0.032 | 5.86 ± 0.18 ^b^ | 0.105 | 5.77 ± 0.17 ^b^ | 0.086 | CTR vs. PGX | 0.008 | 0.015 | 0.079 |
|  | PGX | 39 | 5.78 ± 0.15 ^b^ | <0.001 | 5.82 ± 0.19 ^b^ | 0.001 | 5.97 ± 0.18 ^a,b^ | 0.040 | PSY vs. PGX | 0.866 | 0.867 | 0.418 |
| Glucose | CTR | 45 | 5 ± 0.07 ^a^ | 0.196 | 4.97 ± 0.07 | 0.517 | 4.99 ± 0.07 | 0.202 | CTR vs. PSY | 0.067 | 0.276 | 0.624 |
|  | PSY | 43 | 4.82 ± 0.07 ^a,b^ | 0.466 | 4.86 ± 0.08 | 0.902 | 4.95 ± 0.07 | 0.361 | CTR vs. PGX | 0.019 | 0.118 | 0.449 |
|  | PGX | 39 | 4.76 ± 0.08 ^b^ | 0.049 | 4.8 ± 0.08 | 0.085 | 4.92 ± 0.07 | 0.719 | PSY vs. PGX | 0.538 | 0.621 | 0.785 |

Values are mean ± SEM with baseline as a covariate. Missing values were filled in with LOCF (Last observation carried forward). Different letters in superscript represent significant differences between groups *p* < 0.05. *p* values are within group differences compared to baseline and between groups at 3, 6 and 12 months. CTR (control), PGX (PolyGlycopleX), PSY (psyllium).
